# Supplementary material for: Niche Preference of Escherichia coli in a Peri-Urban Pond Ecosystem
Source: Life (Basel). 2021 Sep 28;11(10):1020. doi: 10.3390/life11101020 (PMC8538306; doi:10.3390/life11101020)
Supplement: Supplementary file 1 [file life-11-01020-s001.zip › Supplementary-life-1367747.pdf]

life

MDPI

Supplementary material

Table S1. Primers used for determining the *uidA* and *mutS* genes, and for phylogrouping.

Primer	Sequence (5' – 3')	Size	Reference
uidA	CATTACGGCAAAGTGTGGGTCAAT (F)<br>TCAGCGTAAGGGTAATGCGAGGTA (R)	658 bp*	[1]
mutS	GGCCTATACCCTGAACTACA (F)<br>GCATAAAGGCAATGGTGTC (R)	596 bp	[1]
chuA	ATGGTACCGGACGAACCAAC (F)<br>TGCCGCCAGTACCAAAGACA (R)	288 bp	[2]
yjaA	CAAACGTGAAGTGTCAGGAG (F)<br>AATGCGTTCCTCAACCTGTG (R)	211 bp	[2]
TspE4	CACTATTCGTAAGGTCATCC (F)<br>AGTTTATCGCTGCGGGTCGC (R)	152 bp	[2]
ArpA	AACGCTATTCGCCAGCTTGC (F)<br>TCTCCCCATACCGTACGCTA (R)	400 bp	[2]
Group E ArpAgpE	GATTCCATCTTGTCAAAATATGCC (F)<br>GAAAAGAAAAAGAATTCCCAAGAG (R)	301 bp	[2]
Group C trpAgpC	AGTTTTATGCCCAGTGCGAG (F)<br>TCTGCGCCGGTCACGCCC (R)	219 bp	[2]

* bp = base pair.

Table S2. Primers used for amplification of virulence genes.

Primers	Sequence (5’ – 3’)	Size (bp)	Reference
stx1	Forward ACACTGGATGATCTCAGTGG Reverse CTGAATCCCCCTCCATTATG	614	[3]
stx2	Forward CCATGACAACGGACAGCAGTT Reverse CCTGTCAACTGAGCAGCACTTTG	779	[3]
eaeA	Forward GTGGCGAATACTGGCGAGACT Reverse CCCCATTCTTTTTCACCGTCG	890	[3]
hlyA	Forward ACGATGTGGTTTATTCTGGA Reverse CTTCACGTGACCATACATAT	165	[3]
STa	Forward GCCTATGCATCTACACAATC Reverse TGAGAAATGGACAATGTCCG	278	[4]
LTb	Forward TATCCTCTCTATATGCACAG Reverse CTGTAGTGGAAGCTGTTATA	480	[4]

* bp = base pair.

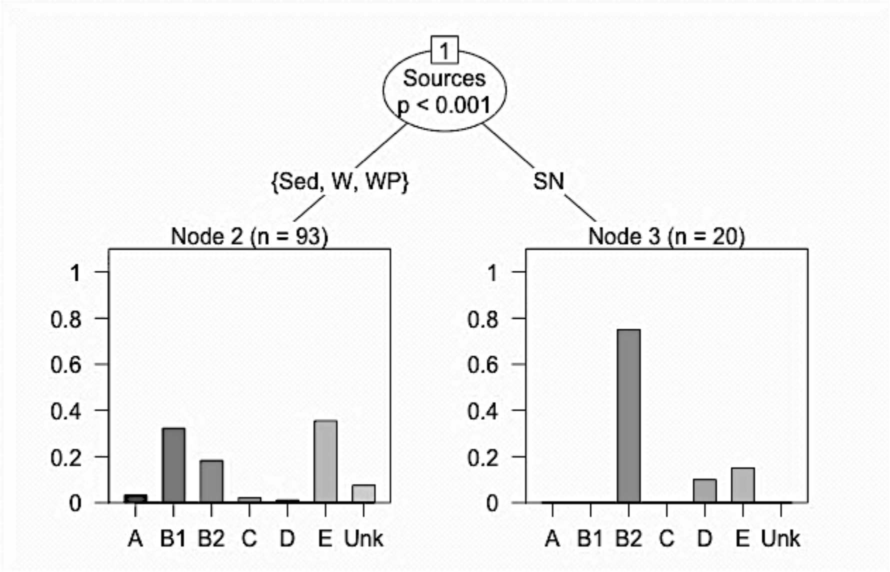

Figure S1. Multinomial log-linear regression analysis of phylogroup distribution of isolates across sample types. Phylogrouping was performed according to the scheme of Clermont et al., 2013. The X axis denotes phylogroups and the Y-axis represents proportion of isolates. Sed – sediment, W – water, WP – water plant, SN – snail.

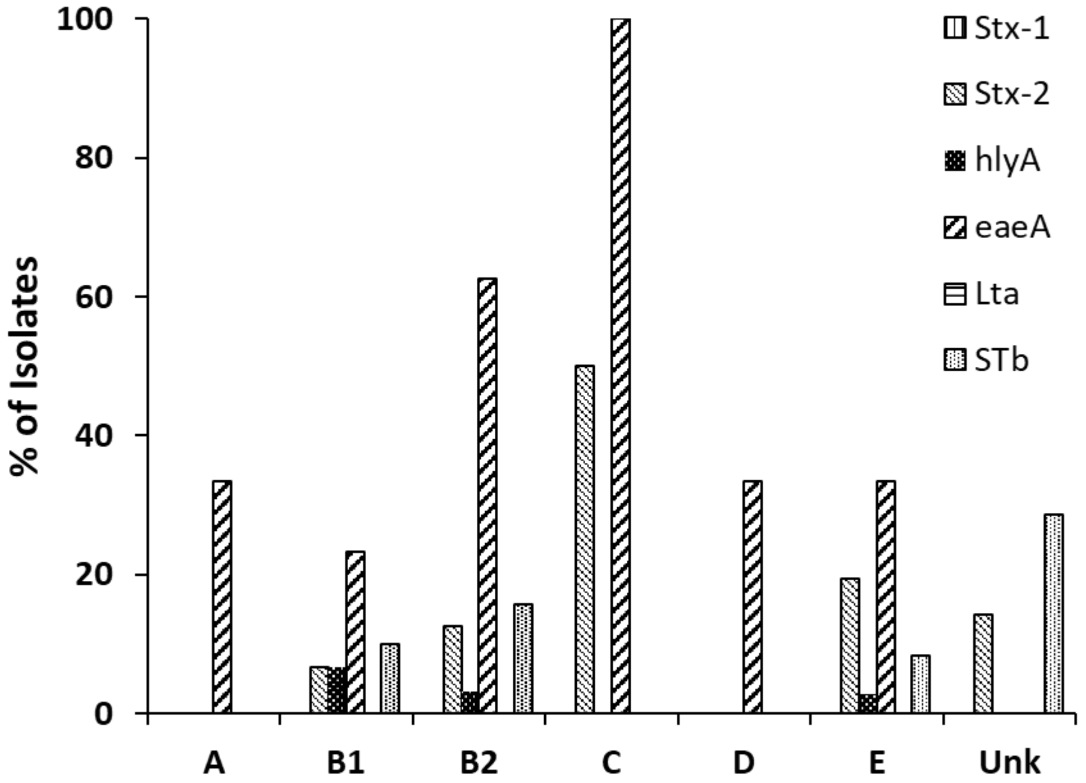

Figure S2. Virulence gene distribution across isolates allocate to phylogroups based on the scheme of Clermont *et al*., 2013. The number of isolates for each phylogroup is given in parentheses on the x axis.

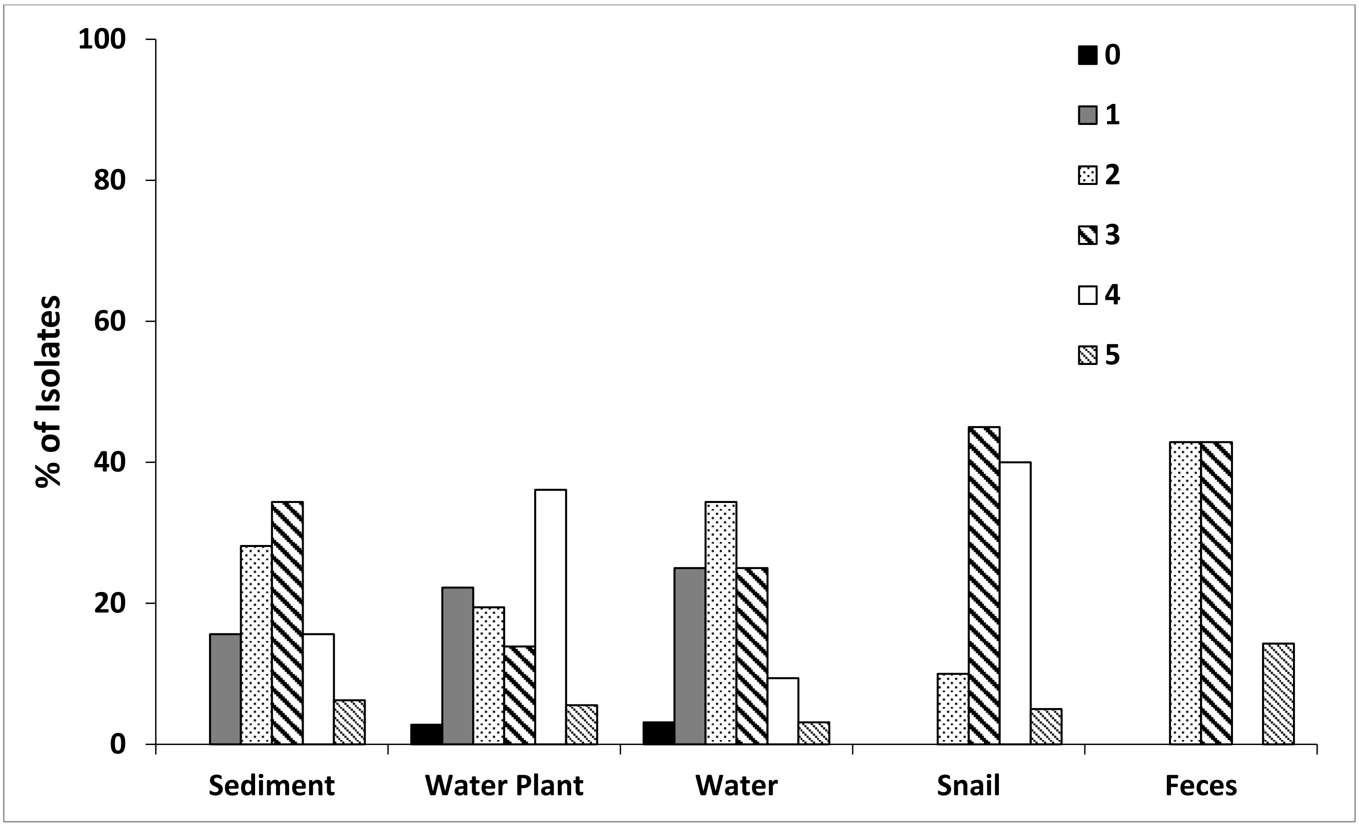

Figure S3. Distribution of sensitive (0) and isolates displaying Intermediate resistance to 1, 2, 3, 4 or 5 antibiotics from the five sampling sites.

1. Walk ST, Alm EW, Gordon DM, Ram JL, Toranzos GA, Tiedje JM, et al. Cryptic lineages of the genus Escherichia. Appl Environ Microbiol. **2009**; 75. doi: 10.1128/aem.01262-09.
2. Clermont O, Christenson JK, Denamur E, Gordon DM. The Clermont *Escherichia coli* phylo-typing method revisited: improvement of specificity and detection of new phylo-groups. Environ Microbiol Rep. **2013**;*5*(1):58–65. doi: 10.1111/1758-2229.12019.
3. Fagan PK, Hornitzky MA, Bettelheim KA, Djordjevic SP. Detection of Shiga-Like Toxin (stx(1) and stx(2)), Intimin (eaeA), and Enterohemorrhagic Escherichia coli (EHEC) Hemolysin (EHEC hlyA) Genes in Animal Feces by Multiplex PCR. Appl Environ Microbiol. **1999**;*65*(2):868–872. PubMed PMID: 9925634.
4. Osek J. Multiplex polymerase chain reaction assay for identification of enterotoxigenic *Escherichia coli* strains. J Vet Diagn Invest. **2001**;*13*(4):308–311. Epub 2001/08/02. doi: 10.1177/104063870101300405. PubMed PMID: 11478602.
